# Pharmacological activity and clinical application analysis of traditional Chinese medicine ginger from the perspective of one source and multiple substances

**DOI:** 10.1186/s13020-024-00969-z

**Published:** 2024-07-12

**Authors:** Cheng Zhang, Anyang Rao, Cui Chen, Yuqing Li, Xiuchi Tan, Jiaxin Long, Xinyue Wang, Junjie Cai, Jiquan Huang, Hua Luo, Chuwen Li, Yuanye Dang

**Affiliations:** 1 Department of Pharmacy, Chengdu University of Traditional Chinese, Medicine, Chengdu, 611137 China; 2 The Fifth Affiliated Hospital, Guangdong Provincial Key Laboratory of Molecular Target and Clinical Pharmacology & NMPA & State Key Laboratory, School of Pharmaceutical Sciences, Guangzhou Medical University, Guangzhou, 511436 China; 3 Hongxing Town Government of Ruoergai County, Aba Tibetan and Qiang Autonomous Prefecture, Sichuan, 624504 China; 4 State Key Laboratory of Quality Research in Chinese Medicine, Institute of Chinese Medical Sciences, University of Macau, Macao, 999078 China

**Keywords:** Ginger, One source and multiple substances, Performance and efficacy, Pharmacological activity, Clinical application

## Abstract

All types of ginger have common fundamental components, although they possess distinct strengths and inclinations when it comes to effectiveness and medicinal applications. Fresh ginger possesses the ability to effectively stimulate movement within the body, alleviate the act of vomiting, induce sweating, and provide relief for external syndromes. Dried ginger possesses both defensive and stimulant characteristics, which effectively raise the internal temperature and enhance the Yang energy. Fresh ginger is more hydrating than dried ginger, highly skilled at heating the Middle-jiao, alleviating pain, halting bleeding, and managing diarrhea. Dried ginger possesses the ability to alleviate coldness when consumed in a heated form, as well as to alleviate diarrhea when consumed in a heated form. It thrives in warm conditions and has a tendency to revert back to its warm nature. The moisture content of baked ginger is inferior to that of dried ginger, but it is highly effective in alleviating pain, bleeding, and diarrhea by warming the Middle-jiao. Ginger charcoal and stir-fried charcoal, produced through carbonization, have excellent heat retention properties and are effective in warming meridians and stopping bleeding. The potency and ability to spread of roasted ginger is less intense compared to fresh ginger, and its moisture content is not as low as that of dried ginger. The medicinal characteristics of this substance are gentle, making it beneficial for alleviating vomiting in patients who are physically frail. Its primary mode of action is on the Middle-jiao. Nevertheless, the main chemical compositions of various traditional Chinese medicines are nearly identical due to their shared base element. Ginger, in particular, possesses a range of pharmacological activities including antioxidant, anti-inflammatory, anti-tumor, anti-bacterial, and anticoagulant properties. However, modern pharmacological research has not fully acknowledged the clinical medicinal value of ginger and consequently, fails to provide accurate guidance for clinical medication. This situation has a negative impact on the contemporary advancement of traditional Chinese medicine (TCM). The research on modernizing ginger is conducted by analyzing and considering the prospects. It is based on Traditional Chinese Medicine (TCM) theory and incorporates the comprehensive perspective of TCM philosophy. In order to modernize ginger, it is essential to have a proper knowledge of the concepts of “recognizing nature by efficacy, homology, and mutual expression of nature and efficacy” and “rationally utilizing modern drug research technology”. By applying these principles, we can construct a bridge towards the advancement of ginger.

## Introduction

Ginger has an extensive historical presence in every country worldwide. It is particularly popular in China. Ginger, a common plant with medicinal and appetizing properties, is extensively utilized in several industries such as food, tea, spices, cosmetics, and more. With a deep-rooted history in the realm of Chinese traditional medicine (TCM) in China, it holds significant potential for use in today’s expansive era of healthcare. Nevertheless, worldwide research on ginger has experienced a progressive reduction in recent years. In therapeutic practice, fresh ginger is commonly utilized, often in combination with different varieties of ginger. Analysis reveals that research on ginger in various nations mostly focuses on studying the primary chemical components of ginger. This narrow approach fails to properly comprehend the holistic perspective of Traditional Chinese Medicine (TCM) philosophy. Consequently, it becomes challenging for modernization research to use TCM theory as a starting point. The chemical compositions of multiple Chinese traditional medicines with the same base element are similar due to the specificity of one source and multiple substances. Defining their pharmacological activities based on a single or a class of major chemical compositions cannot adequately highlight the unique effects of each component of ginger. Therefore, it is crucial to consider the holistic view of Traditional Chinese Medicine (TCM) theory in the modernization research of TCM's one source and multiple substances. The objective of this study is to determine the origin of ginger and conduct a comparative analysis of its performance, efficacy, pharmacological activity, and clinical application. Additionally, this study aims to share Chinese wisdom in the field of healthcare by examining ginger from both Traditional Chinese Medicine (TCM) theory and modern pharmacological activity. The goal is to enhance researchers’ understanding of the origin of ginger in TCM and its distinctive uses, provide a theoretical foundation for studying various processed ginger products in clinical medicine, and promote further exploration of ginger. Simultaneously, the research provides a fair analysis of the global research trend of ginger through the lens of patent applications. This analysis can also offer research direction and theoretical guidance for the comprehensive advancement of ginger.

## Origin of ginger

The study of ginger originated from the “Huangdi Neijing” (The Inner Canon of Huangdi) [1], and it was initially documented as a medicinal herb in the “Shennong Ben Cao Jing” (Shennong’s Herbal) [2]. Numerous records and extensive research have been conducted on ginger, encompassing detailed investigations into its processing methods and pharmaceutical standards. It has been established that ginger, dried ginger, and powdered ginger are three distinct forms derived from the same herbal source. Ginger has been included in the Chinese Pharmacopoeia (2020 Edition), providing comprehensive information on its properties (Table 1) [3].

**Table 1:** ^Corresponding processing methods and drug standards for fresh ginger, dried ginger, and baked ginger in the new era^

Processed varieties	Processing method	Content requirements
Fresh ginger	1. Remove impurities from fresh ginger; 2. Wash it thoroughly; 3. Cut it into thick slices	A. Detection using high performance liquid chromatography B. The content of 6-gingerol ≥ 0.050% C. The content of total ash ≤ 7.0%
Dried ginger	1. Remove impurities from it; 2. Soak it slightly; 3. Wash it thoroughly; 4. Moisten it thoroughly; 5. Cut it into thick slices or pieces, and dry	A. The content of volatile oil ≥ 0.8% (ml/g) B. Detection using high performance liquid chromatography C. Calculated as a dried product, the content of 6-gingerol ≥ 0.60% D. The content of moisture ≤ 19.0% E. The content of total ash ≤ 6.0%
Baked ginger	Take dried ginger and stir fry it according to the method (General Rule 0213), heat it in sand until it is bulging, with a brownish surface	A. Detection using high performance liquid chromatography B. The content of 6-gingerol ≥ 0.30% C. The content of moisture ≤ 12.0% D. The content of total ash ≤ 7.0%

Fresh ginger, known as the “the holy medicine of vomiting” in TCM, is the fresh rhizome of the *Zingiber* *officinale* Rose. It has a pungent taste and a slightly warm nature, and is beneficial to the lungs, spleen, and stomach meridians. Fresh ginger is collected from ginger roots in autumn and winter, washed for use of raw materials, and fresh for medicinal use, also known as purple ginger, garlic or old ginger. It was originally attached to dried ginger, and then gradually separated out. Zhang Zhongjing clearly proposed using ginger as medicine in “Shang Han Za Bing Lun” (Treatise on Febrile and Miscellaneous Diseases) [4], such as Guizhi Shengjiang Zhishi Decoction and Shengjiang Xiexin Decoction. In addition, Zhang Zhongjing also uses fresh ginger juice as medicine, including Shengjiang Banxia Decoction, “boiling Pinellia ternata, taking two liters, collecting fresh ginger juice for use”, and Ganjiang Renshen Banxia Pill, “fresh ginger juice paste is used as pill, which is prepared by pounding fresh ginger juice into medicine”.

Dried ginger is the dry rhizome of ginger in Zingiberaceae, which is pungent and hot in nature, and acts on spleen, stomach, heart and lung meridians. Dried ginger was first contained in “Shennong Ben Cao Jing” [2], which states that “those who are white, clean, and strong are good”, hence it is also known as white ginger. According to ancient books, there are two ways to prepare dried ginger: one is to “collecting roots in autumn, washing them in long flowing water, and sunning them to make dried ginger” [5], and the other is to “making dried ginger by soaking it in water for three days, removing the peel and placing the ginger in flowing water for six days, and then scraping the peel and drying the ginger, and then storing the ginger in a porcelain jar for three days” [6].

Baked ginger (Zingiberis Rhizoma Praeparatum) is the processed product of dry rhizome of the perennial herb in Zingiberaceae, which is pungent and hot in nature and acts on spleen, stomach and kidney meridians. The use of baked ginger was first described in “Jin Kui Yao Lue” (Prescriptions from the Golden Cabinet) and is listed separately as a single medicine in “Depei Bencao” [7]. Baked ginger, also known as black ginger, is made from dried ginger as the raw material and processed with processing methods. The processing method includes: “pharmacists using dried ginger to prepare black ginger, which is called baked ginger” [8], and “Depei Bencao” states that “baked ginger is the one that adopts dried ginger water to clean and roast yellowed parts of the ginger” [7].

In Chinese medicine books, but not in the pharmacopoeia, there are also ginger charcoal and simmering ginger. Ginger charcoal has a bitter and astringent taste, a warm nature, and is divided into the meridian and blood of the spleen meridian of foot taiyin. It is a processed product of dried ginger, and in clinical applications, ginger charcoal is often classified as a mixture of baked ginger, but there is a fundamental difference between the two drugs. There are significant differences in the processing methods and degree between ginger charcoal and baked ginger. In the processing of baked ginger, it is better to stir fry ginger with sand. Blanching method has the advantages of evenly heating and homogeneous processing degree. The method involves the use of many auxiliary materials, has a short processing time, less carbonization, and pays attention to the swelling degree of the medicine, aiming to remove part of volatile oils [9]. Ginger charcoal, on the other hand, is preferred for plain-frying, with high firepower for concocting and much charring of the product, focusing on the color of the drug, with the intention of stir-frying the charcoal to preserve its nature [10]. According to “Taiping Huimin Heji Ju Fang” (Prescriptions of the Bureau of Taiping People's Welfare Pharmacy), up to the Song Dynasty, in addition to the “processing” for dried ginger mentioned in the “Taiping Shenghui Fang”, there is another “baked charcoal”, i.e. ginger charcoal [11]. Therefore, it can be assumed that ginger charcoal has been another processed product of dried ginger since the Song Dynasty. Miao Xiyong in the Ming Dynasty pointed out that: “If the patient suffers from postpartum blood deficiency and fever, in order to stop bleeding, we should stir-fry the charcoal until black, process the charcoal for use in warming the Middle-jiao, disperse cold evil, regulate lung qi and stop vomiting” [12]. In “Bencao Chongyuan” [8], it is pointed out that “later generations referred to the process of preparing dried ginger until black as baked ginger”. The taste of ginger was originally pungent, but after it is processed, the pungent taste was slightly reduced. If it is processed too much, the essence did not exist, and this was called ginger charcoal. Its taste was slightly bitter but not pungent, its quality was light and not solid, and it was not as functional as baked ginger. Therefore, baked ginger and ginger charcoal can be considered as two different processed products of dried ginger. To sum up, although baked ginger and ginger charcoal are processed products of dried ginger, there are similarities in processing, but there are fundamental differences between them in processing degree, effect, price and clinical application, so we should strictly distinguish medicinal use in management and usage. Roasted ginger has a pungent taste, warm nature, and can enter the lungs and stomach meridians. It is one of the medicinal methods for ginger. According to research, in ancient times, the ginger used for roasted ginger was not unified. In “New Compilation of Materia Medica”, dried ginger was used [13], and in “Depei Bencao Shengjiang”, fresh ginger was used [7]. At present, roasting of ginger excerpted from Anthology of Chinese Herb of Whole Nation [14] means that Zingiber officinale Rosc is washed, freshly used, wrapped in papyrus, soaked in clear water, and simmered directly in the fire until the papyrus is burned black and the ginger is cooked to the right degree of softness; Or directly set on fire and grilled until cooked.

**Fig. 1:**
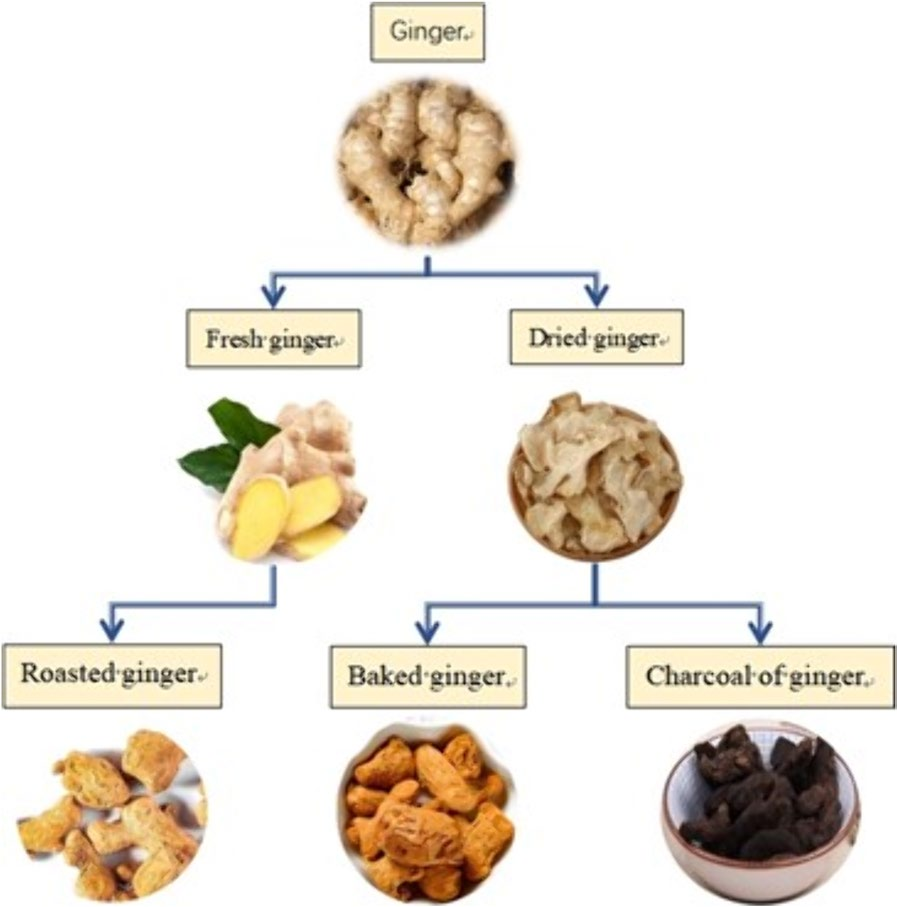
All kinds of gingers and their connections

The pharmacological effects of traditional Chinese medicine are often manifested through the use of herbal decoctions. Pharmacological effects are the external manifestations of medicinal properties, and through understanding the pharmacological effects of TCM, we can comprehend their medicinal properties, which is known as the principle of “recognizing properties through effects”. Pharmacological effects and medicinal properties are essentially the internal and external manifestations of the unified attributes of TCM. Internally, they are described abstractly as the Four Qi and Five Tastes, while externally, they are recorded as clinical efficacy, representing the unity of properties and effects. Medicinal properties are the expression of pharmacological effects in TCM theory, while pharmacological effects are the observable manifestations of medicinal properties in practical use, thus representing the mutual reflection of properties and effects. Therefore, modern research on TCM advocates a holistic approach, starting from the principles of "recognizing properties through effects, unity of properties and effects, and mutual reflection of properties and effects." This approach involves studying the pharmacological effects in relation to the main chemical components and clinical research in modern pharmacology, enriching our understanding of the properties of ginger from a modern perspective, and also supplementing areas that have not yet been recognized from the standpoint of modern scientific technology (see Fig. 1).

Based on the basic traceability, connecting with the image thinking of understanding the medicinal properties of TCM, starting from “recognizing nature by efficacy, homology and mutual expression of nature and efficacy”, we can also know the origin of ginger by studying the main chemical components of modern TCM. At present, the research on TCM is mostly based on effective parts or effective components extracted from them, and different clinical efficacies are determined according to their main medicinal effective ingredients. Analysis of the main chemical components of various kinds of ginger indicates that in addition to nutrient substances such as cellulose, starch, and fat, the functional components of various kinds of ginger mainly include volatile oil, gingerol, diphenylheptane, and active polysaccharides [15]. However, different processing methods lead to some differences in the effective components contained in ginger. According to the determination requirements of Chinese Pharmacopoeia (2020 Edition) [3], we can also see that some chemical components of ginger and its processed products have obvious changes. By comparing the volatile oil components of various kinds of ginger, it was found that there is a total of 43 chemical components, among which fresh ginger, dried ginger, and ginger charcoal each contain 27 types of chemical components, while baked ginger contains 24 chemical components [16]. From fresh ginger to dried ginger, baked ginger, and ginger charcoal, with the deepening of processing methods and degrees, the content of 6-shogaol gradually increases, while the content of 6-gingerol gradually decreases. After stir-frying, zingerone appears in baked ginger and ginger charcoal, with baked ginger having the highest content [17]. There are also significant differences in the content of active polysaccharides in various ginger varieties, with dried ginger being the most abundant, followed by baked ginger and ginger charcoal, and fresh ginger being the least abundant [18]. In addition to the change of chemical composition content, processing will also lead to the addition of chemical composition. For example, compared with other processed products, 3,7,11-trimethyl-1,6,10-dodecatrienal,3,9(11)-diene-10-peroxide, cubebene, eudesma-4,11-dlene and β-red myrrh alcohol are newly produced in baked ginger [19]. In summary, starting from the main chemical components, there are also differences in the overall efficacies of various kinds of ginger.

## Pharmacological effects and clinical studies of ginger

### Cognition of efficacy based on TCM theory system

**Fig. 2:**
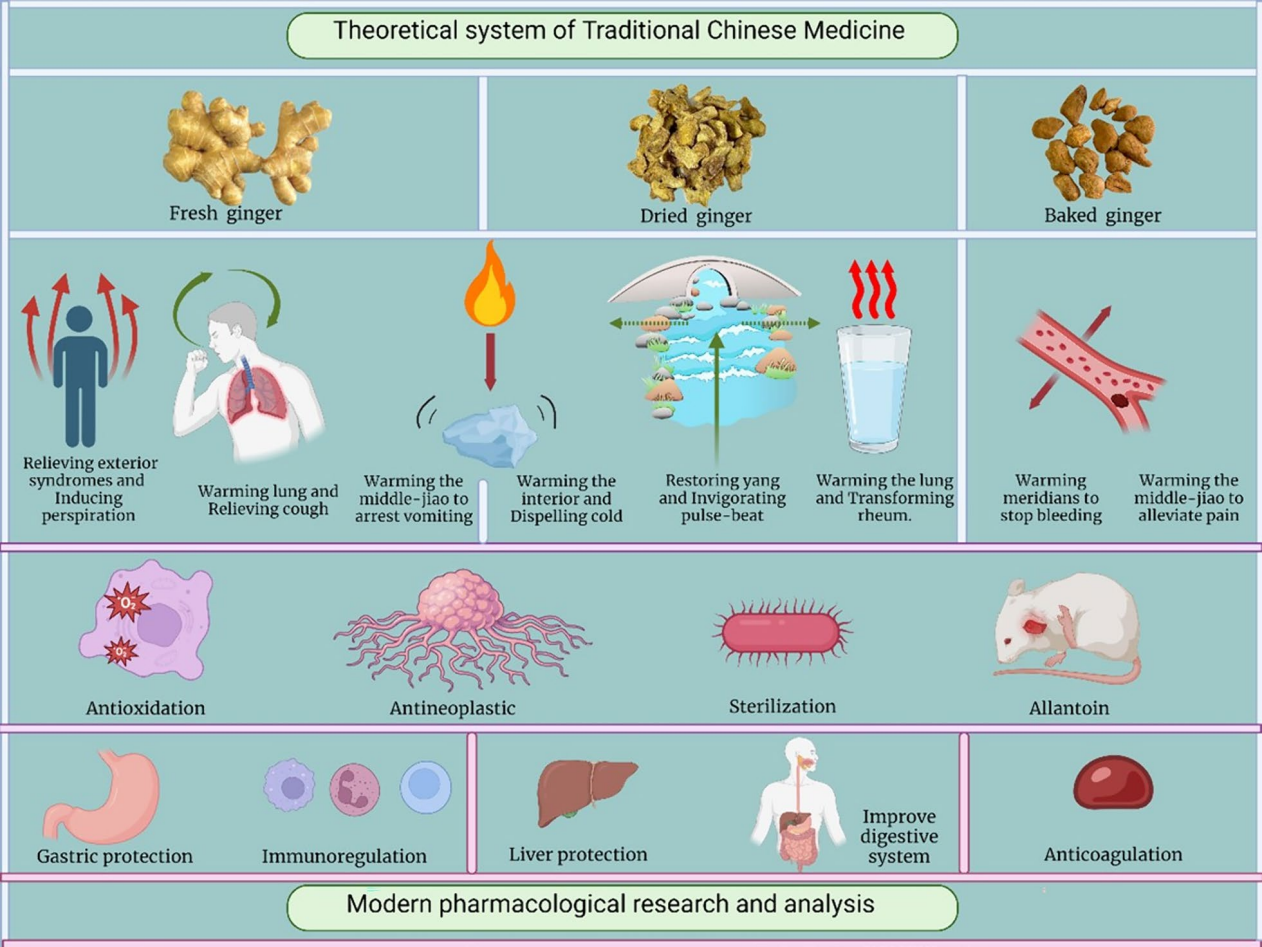
Visually represents the medicinal properties of fresh ginger, dried ginger, and baked ginger, based on the analysis of “identifying properties by effects, homology of properties and effects, and mutual expression of properties and effects”, as well as “modern drug research”

The book “Mingyi Bie Lu” [20] states that fresh ginger has the ability to balance the body’s external conditions and promote sweating, which helps eliminate wind-cold-damp microorganisms that attack the lung’s immune system. Consequently, fresh ginger possesses the ability to alleviate symptoms such as headache, nasal congestion, cough, and nausea that are induced by colds and febrile disorders. The book “Bencao Wenda” [21] states that fresh ginger’s warming power is specifically effective in treating cold-dampness under wind-cold circumstances. The inhibitory impact of this substance can extend from the digestive tract to the respiratory tract, thereby reducing nausea and vomiting. Additionally, it has the ability to remove detrimental elements that lead to the development of diseases from the body’s exterior, which is demonstrated by perspiration and increased body temperature, as depicted in Fig. 2. Ginger’s warming properties can specifically alleviate respiratory and pulmonary problems, as documented in the “Yaoming Lun” [22]. Ginger can be used to treat illnesses characterized by vomiting caused by numerous circumstances and pathogenic agents.

Dried ginger is classified as a warm internal medicine and has the properties of heating the middle, eliminating cold, enhancing the flow of yang qi, warming the lungs, and converting phlegm. In comparison to fresh ginger, dried ginger possesses a higher level of concentration and desiccating properties, rendering it more appropriate for the expulsion of moisture, elimination of cold, stimulation of the lungs, and alleviation of cough and wheezing in the central region of the body without impacting the superficial regions. Dried ginger exhibits rapid action and possesses potent medicinal properties, as depicted in Fig. 2, wherein the augmented velocity of the river water can promptly penetrate stones. Dried ginger has a fast and powerful impact that can swiftly alleviate obstructions caused by cold and dampness in the body’s energy pathways, enabling the vital energy to rise and effectively restore vitality and reverse a state of collapse. In the field of clinical medicine, this treatment is particularly efficient for severe and critical situations characterized by a strong dislike or avoidance of cold temperatures. The book “Bencao Qiuzhen” [23] mentions that when there is a deficit of warmth in the stomach and the original yang is about to be depleted, combining it with Fuzi (Aconiti Radix lateralis preparata) will help replenish yang and have immediate results. Therefore, it is commonly said that “dried ginger lacks spiciness without the addition of Fuzi”.

Baked ginger is a hemostatic drug, which has the functions of warming meridians to stop bleeding and warming the Middle-jiao to alleviate pain. The pungent and dry nature of baked ginger is weaker than that of dried ginger, and its power of warming the interior is not as swift and violent as that of dried ginger. However, the effect of baked ginger is gentle and long-lasting, and the drug has a wide range of properties and rapid action. Baked ginger can dry the cold and dampness of the spleen and stomach, remove the cold and stuffy sensation in the navel and abdomen, warm the heart qi, warm the liver and spleen meridians, and restrain the excessive blood leakage. Therefore, the baked ginger’s power is more concentrated in the blood phases of middle and lower jiaos, and it is good at warming the Middle-jiao to alleviate pain, relieving diarrhea, and warming meridians to stop bleeding. Both of them are processed products of dried ginger, and the nature and efficacy of ginger charcoal is similar to that of baked ginger. The drug has a wide range of properties and rapid action, and it has good retention and is good at warming channel for arresting bleeding, and warming the spleen and anti-diarrhea. Miao Xiyong pointed out in “Bencao Jingshu” [24] that “dried ginger that stir-fried until black can attract various hematic tonics into the yin, and when blood is replenished, yin is generated and heat is reduced, and there will be no bleeding”. But there is a fundamental difference between the two. The preparation methods of baked ginger and ginger charcoal are different. Baked ginger is intended to remove some volatile oils, and it tends to warm the Middle-jiao, while ginger charcoal is intended to stir fry charcoal to preserve its natures, and it tends to stop bleeding. Therefore, although there are overlapping pharmacological effects, each has its own strengths. National Standard for the Processing of Traditional Chinese Medicine clearly distinguishes the two [25]: “baked ginger is used to warm the interior and dissipate cold, and is used for deficiency-cold of the spleen and stomach, abdominal pain, vomiting, and diarrhea. Ginger charcoal can warm meridians and stop bleeding, and is mainly used to treat vomiting, epistaxis, metrorrhagia and blood loss due to yin deficiency”. Ginger can be used for medicinal purposes by simmering. Roasted ginger appears as a common method of fresh ginger medicine, and has the efficacy of regulating the middle warmer and stopping vomiting. Compared with fresh ginger, conventional medicine believes that simmering can reduce the divergence of fresh ginger, increase the principle of yang-heat, help the spleen and stomach in transportation and transformation, and make the Tai Yin damp soil warm and move, thus enhancing the functions of warming the Middle-jiao to arrest vomiting. However, due to limited application, there are few studies.

Based on the theoretical system of TCM on one source and multiple substances of ginger, it can be seen that there are differences in effect or efficacy among different kinds of ginger, and it is not advisable to generalize in drug management and clinical application. All kinds of ginger have their own unique utilities and have great research value. As illustrated in Fig. 2, according to traditional Chinese medicine theory, the therapeutic effects can be expressed in the following ways: (1) Sweating to relieve symptoms: The arrow signifies the movement of pathogenic factors from the body to the skin's surface through sweating; (2) Warming the lungs and relieving cough: The green arrow indicates an improvement in the respiratory condition; (3) Warm to stop vomiting, warm to dispel cold: The gradual warming process is likened to heating cold water over a low fire, causing the cold air to dissipate like water vapor; (4) Huiyang Tongmai: The medicine's rapid effect is likened to the forceful impact of river water, swiftly breaking through blockages in the meridians caused by cold and dampness, allowing Yang Qi to surge and restore vitality. It is particularly effective in severe and emergency cases; (5) Warming the lungs and transforming the drink: A powerful gust of hot wind melts away icy water, symbolizing the transformation of coldness; and (6) Warming and relieving pain, warming menstruation and stopping bleeding: These effects are achieved by dilating blood vessels and reducing pressure.

### Analysis based on modern pharmacological research

In recent years, with the in-depth study of ginger by scholars at home and abroad, modern pharmacological analysis of ginger has been carried out, but the research and analysis of baked ginger, ginger charcoal and roasted ginger, which are little known, are relatively lacking, mainly focusing on the pharmacological research of fresh ginger and dried ginger. Studies have found that fresh ginger can not only stop vomiting, but also have anti-oxidation, anti-inflammatory, anti-tumor and bactericidal effects, and stomach protection, metabolism regulation, immunity regulation, anticoagulation and blood lipid and blood glucose reduction effects; Dried ginger has anti-inflammatory, antibacterial, anti-oxidation and anti-tumor functions, and liver protection, digestive system improvement, and blood system improvement effects. In addition, very few studies have also shown that baked ginger can be anticoagulant, anti-inflammatory, anti-tumor and anti-oxidation; Ginger charcoal can warm meridians, stop bleeding, resist oxidation, resist inflammation and relieve pain [26]. There is currently a lack of corresponding researches on roasted ginger. In summary, it can be seen that there are many overlapping pharmacological effects among different kinds of ginger, mainly anti-oxidation, anti-inflammatory, anti-tumor, anticoagulant, and corresponding analgesic and antibacterial effects. Based on modern pharmacological research, this paper will conduct an in-depth analysis of the pharmacological effects of various ginger varieties.

Presently, the main emphasis in pharmacological study lies on fresh ginger and dried ginger, whereas there is a dearth of research and analysis on less familiar processed ginger items such as fried ginger, charred ginger, and stewed ginger. Research has shown that fresh ginger possesses not only antiemetic characteristics, but also demonstrates antioxidant, anti-inflammatory, anti-tumor, antibacterial, gastroprotective, metabolic-regulating, immunomodulatory, anticoagulant, and hypolipidemic benefits. In contrast, dried ginger has anti-inflammatory, antibacterial, antioxidant, anti-tumor, hepatoprotective, digestive system-enhancing, and hematological system-improving effects. Preliminary studies indicate that fried ginger possesses anticoagulant, anti-inflammatory, anti-tumor, and antioxidant qualities, whereas charred ginger demonstrates characteristics such as enhancing blood circulation, preventing bleeding, antioxidant activity, anti-inflammatory benefits, and pain relief [28]. Nevertheless, there is presently a dearth of research on the effects of stewed ginger. In general, different varieties of ginger have similar pharmacological benefits, particularly in terms of their ability to act as antioxidants, reduce inflammation, inhibit tumor growth, prevent blood clotting, and provide pain relief. Additionally, ginger also possesses antibacterial capabilities. Figure 2 presents a comprehensive examination of the similarities and distinctions in the pharmacological impacts of different varieties of ginger, as determined by contemporary pharmacological research.

### Comprehensive pharmacological effect of fresh ginger and its research situation

Fresh ginger has a special vomiting stopping effect, mainly because the gingerol compounds it contains plays a major role in reducing the stimulation of the vomiting center and reducing the release of related neurotransmitters. After research [27], it was found that its mechanism of action may be that the four gingerol components in fresh ginger, i.e. 6-gingerol, 8-gingerol, 6-shogaol, and 10-gingerol, and highly polar glycosides, reduce the expression of neurotransmitter system related receptors such as 5-HT, dopamine, and substance P in the central and peripheral nervous regions, alleviate 5-HT3-stimulated vomiting-inducing chemoreceptor (CTX) and M3 choline receptors, and thus produce antiemetic effects. In addition, research has found that the aglycones of the four gingerol glycosides in fresh ginger all present two or more hydroxyl groups on the alkyl chain and do not contain any other substituents. It is speculated that this type of gingerol is more effective in entering the human body due to its combination with carbohydrate components, and has an anti-emetic effect in the body.

Fresh ginger species contain gingerol with strong antioxidant activity. 6-Gingerol, 8-gingerol, and 10-gingerol can effectively eliminate 1,1-diphenyl-2-picylhydrazyl (DPPH), superoxide, and reactive hydroxyl radical (OH) [28]. Its key chemical basis lies in guaiacol structure and β-hydroxyketone structure, and its oxidation resistance is higher than that of antioxidants such as butyl hydroxyanisole, dibutyl hydroxytoluene and vitamins. It was found that [29], the contents of malondialdehyde (MDA) and GSSG in plasma of chickens treated with gingerol were significantly decreased, the value of GSSG/GSH was significantly increased, and the total antioxidant capacity was significantly improved. Among them, 6-gingerol acts on cell membrane lipids, cytochrome, superoxide radicals, hydroxyl radicals, and DPPH radicals, inhibiting the peroxidation, DNA fragmentation, cytochrome oxidation, and iron ion reduction of red blood cell membrane lipids. Its inhibition rate of DNA oxidation is as high as 91.26%, and the clearance rate of superoxide radicals, hydroxyl radicals, and DPPH radicals exceeds 80% [30]. Fresh ginger extracts such as 6-shogaol and fresh ginger essential oil also have antioxidant capacity. 6-shogaol acts on heme oxygenase-1 (HO-1) and Nrf2, increasing the mRNA and protein expression of HO-1 and Nrf2, reducing the production of reactive oxygen species (ROS) in cells, and inhibiting oxidative damage in L6 skeletal muscle cells [31].

Fresh ginger also has anti-tumor activity, with its main active ingredients including gingerol and shogaol. The potential mechanisms include regulating cancer related signaling pathways, inducing cancer cell apoptosis, and inhibiting cancer cell growth and reproduction. According to research [32], fresh ginger extract promotes apoptosis by reducing the expression of genes related to Ras/extracellular signal-regulated kinase (ERK) and PI3K/Akt pathway. Notably, although there were few studies on fresh ginger polysaccharide, its anti-tumor ability was not inferior to that of other active ingredients, and it could also promote apoptosis, block cells in G0/G1 phase, up-regulate the expression of antibody genes (Bax, Fas, FasL), caspase 3, and cell cycle coordination factors (p21, p53), and down-regulate the expression of B cell lymphoma/leukemia-2 (leukemia-2, Bcl-2) oncogene. 6-Shogaol may be more significant than 6-gingerol and 6-paradol in reducing cell survival rate and inducing apoptosis of human and mouse prostate cancer cells, and it mainly plays a role by inhibiting signal transduction and transcription activator 3 (STAT3) and NF-κB signal transduction [33].

Fresh ginger volatile oil and gingerol also have good anti-inflammatory activities, among which 6-paradol, 6-shogaol and 8-gingerol have strong anti-inflammatory activities, which can inhibit the inflammatory reaction by increasing the expression of anti-inflammatory factors, reducing the expression of inflammatory factors and pain-causing factors and blocking the activation of inflammation-related pathways [34]. Among them, cedrol in the fresh ginger volatile oil improved inflammatory cell infiltration and synovial hyperplasia by blocking the phosphorylation pathways of ERK/MAPK and p65/NF-κB signaling pathways [35]. In addition, 6-gingerol can inhibit the systemic inflammation of obese zebrafish by increasing the anti-inflammatory cytokine IL-10 and activating c-Jun N-terminal kinase (JNK), and can also decrease the expression levels of pro-inflammatory cytokines IL-1β, IL-6 and inducible NO synthase, and down-regulate the signal pathway of serine-threonine Akt-MTOR signal transducers and activators of transcription 3 (STAT3), so as to inhibit neuroinflammation mediated by microglia [36]. The anti-inflammatory effect of 6-gingerol may also be related to inhibiting the activation of macrophages and neutrophils and affecting the migration of monocytes and leukocytes [37]. In addition, some studies have shown that zingerone can reduce the expression of inflammatory factors such as PGE, NO, COX-2 and malondialdehyde (MDA) (oxidative stress index) and play an anti-inflammatory role [38].

Fresh ginger contains fresh ginger oil, gingerol, fresh ginger flavonoid, and fresh ginger polyphenol, all of which have varying degrees of antibacterial activity. Fresh ginger oil has a good inhibitory effect on fungi and bacteria, especially penicillium, Aspergillus Niger, Shigella dysenteriae and Bacillus thuringiensis, and the fresh ginger oil extracted by Soxhlet extraction method has a good inhibitory effect on *Escherichia* *coli* [39]. Gingerol has a strong inhibitory effect on *Helicobacter* *pylori* [40], presumably because 6-gingerol interacts with some enzymes needed for the growth of *Helicobacter* *pylori* to inhibit its growth and death. In addition, it was found that 6-gingerol, 6-paradol, and 6-shogaol could restrain the appearance of ergosterol, destroy the integrity of fungal cell membrane and lead to the death of fungi [41]. The flavonoids from Rhizoma Zingiberis Recens all had a certain inhibitory effect on various bacteria, and their inhibitory intensity on the bacteria was in the order of *Bacillus* *subtilis* > *Aspergillus* *niger* > Penicillium > *Escherichia* *coli*, and the minimum inhibitory concentration on the above bacteria was in the order of *Escherichia* *coli* (10%) > Penicillium (5%) > *Aspergillus* *niger* (3%) > *Bacillus* *subtilis* (1.5%) [42]. The antibacterial rate of fresh ginger polyphenol to *Escherichia* *coli* reached 75% [43]. At present, the antibacterial mechanism of fresh ginger needs further study and improvement.

Studies at home and abroad have shown that fresh ginger can play an anticoagulant role by resisting platelet aggregation, changing blood flow velocity and reducing blood viscosity. Lee et al. [44] found that, zingerone, the active ingredient of fresh ginger, can inhibit platelet aggregation by reducing the time of activating partial thromboplastin, and play an antithrombotic and anticoagulant role. Wang et al. [45] showed that fresh ginger polysaccharide prolonged prothrombin time (PT) and activated partial thrombin time (ATPT) and inhibited coagulation of endogenous and exogenous pathways. Therefore, fresh ginger polysaccharide could be used as a natural anticoagulant and therapeutic agent. In addition, the fresh ginger alcohol extract was proven to be able to to significantly inhibit adenosine diphosphate-mediated platelet aggregation, possibly due to the inhibition of arachidonic acid-induced platelet aggregation by gingerol [46].

Gingerol compounds from fresh ginger can reduce the cholesterol content of the body by inhibiting cholesterol synthesis and increasing exogenous intake of cholesterol. Gingerol compounds from fresh ginger can also reduce blood lipid and glucose by reducing adipocyte differentiation, increasing fatty acid oxidation capacity, or inhibiting fatty acid synthesis, and reducing fatty acid and lipid accumulation of the body. The study found [47] that, by regulating nuclearfactor erythroidderived 2-like 2 (Nrf2), 6-gingerol can also reduce the levels of advanced glycation end products (AGEs) and carboxymethyl lysine in the liver tissue of C57BL/6 mice fed with high-fat diet, improve the reduced glutathione, r-glutamyl cysteingl+glycine/ glutathiol (GSH/GSSG) value and reduce the complications caused by diabetes. Active ingredients such as gingerol have regulatory effects on humoral, cellular, and intestinal immunity [48], which can enhance the body's immune system, prevent and treat various immune disorders. Zhang Lihua et al. [49] found through network pharmacology that three active components of fresh ginger (6-gingerol, 8-gingerol and 10-gingerol) acted on 18 important target proteins in COVID-19, and the biological process enrichment mainly focused on inflammatory reaction and immune response, such as leukocyte differentiation, hematopoietic or lymphoid organ development, response to cytokines, immune system development, T cell activation and H cell activation.

### Comprehensive pharmacological effect of dried ginger and its research situation

Experimental results showed that the main antioxidant components of dried ginger are zingerone, gingerol, shogaol and other compounds. Yang et al. [31] used H_2_O_2_ and azalea alcohol to induce oxidative stress in normal human primary melanocytes as a model, demonstrating that 6-shogaol can alleviate the damage of H_2_O_2_ and azalea alcohol to human primary melanocytes by upregulating the expression of antioxidant enzymes heme oxygenase-1 (HO-1) and quinone oxidoreductase-1 (Nqo1), activating Nrf2-antioxidant response element (Nrf2-ARE) pathway. Li Jiahui et al. [50] adopted selective reaction/multiple reaction monitoring (SRM/MRM) to conduct qualitative and quantitative analysis of the active components in dried ginger extracts and measure the antioxidant and in vitro uric acid reduction activities of Rhizoma Zingiberis extract and its active components. Dried ginger extracts have significant antioxidant effect and strong uric acid reduction activity. Li Shumei et al. [51] studied the effects of different temperature and pH on free radical scavenging ability of ginger flavonoid compounds from dried ginger by DPPH method, and also showed good antioxidant activity.

Dried ginger is the essential drug of warming the Middle-jiao, and its main active components are gingerol and shogaol, with 6-gingerol, 6-shogaol and 8-gingerol as its representative components. It has been successively reported by domestic and foreign journals that it has good natural anti-tumor effect and anti-inflammatory and antibacterial activity. John F. Lechner[52] thinks that its bioreactive constituents negatively affect carcinogenesis of multiple cell types via multiple molecular mechanisms. Ginger species have the property to bind to and inhibit the activity of cytoplasmic proteins. The mechanism(s) of this selectivity for gingerols, shogaols, and paradols appears to be differential activation of the glycosylated metabolites by higher levels of β-glucuronidase in the tumor cells, whereas zerumbone may discriminate because of cell-type-specific differential up-take. Geng Shengnan [53] screened a variety of active ingredients in dried ginger according to the two criteria of “five principles of drug-like properties” and “oral bioavailability” ≥30%, predicted the anti-tumor targets of various active ingredients and their corresponding drugs, and concluded that there were 52 effective active ingredients with good drug-like properties, oral and absorptive activities in dried ginger molecules, and 101 corresponding effective targets. There were 40 anti-tumor metastatic targets and 10 core targets in total. Pharmacological studies have shown that dried ginger and its monomer components have therapeutic effects on breast cancer, cervical cancer, gastric cancer, colorectal cancer and other cancers. Among them, 6-gingerol has an obvious effect on the S phase of the proliferation cycle of human cervical cancer Hela cells (HeLa) [54]. It is speculated that it may inhibit cell proliferation by affecting the replication of genetic substances and the formation of nucleosomes.

The dried ginger shows good anti-inflammatory activity, and the dried ginger extract contains a large amount of gingerols and diphenyl isoheptanes. The diphenylheptane A contained in it can directly pass through a type 2 receptor protein (COX-2) receptor which affects the activity of lipoxygenase, thereby significantly reducing prostaglandin inflammatory mediators produced by metabolic enzyme processes such as arachidonase in vivo, and can further effectively enable fat to be incorporated into lysophospholipids from 20:4 fatty acid chains, thereby greatly increasing the content of glycerophospholipids which originally only contains about 20:4 fatty acid chains and playing a significant anti-inflammatory role [55]. In addition, dried ginger also has antibacterial activity. Li Jiaqi et al.[56] studied the antibacterial activity of the ethanol extracts of fresh ginger and dried ginger through in vitro antibacterial experiments. Data show that both ginger and dried ginger have strong inhibitory effects on Staphylococcus aureus, and have certain inhibitory effects on Pseudomonas aeruginosa and Bacillus subtilis, while ginger has no inhibitory effects on *Escherichia* *coli*, and dried ginger has weak inhibitory effects.

Dried ginger can stop bleeding, generate blood, and promote blood circulation. In terms of hemostasis, dried ginger can warm the spleen, harmonize the liver and stomach, stop bleeding, warm the stomach, descend qi, stop bleeding, warm yang, astringe the intestines, stop bleeding, warm channel and expelling cold, and stop bleeding. On the aspect of blood production, dried ginger can induce drugs to enter the blood and generate blood, or induce drugs to enter the qi and generate blood. On the aspect of blood circulation, the spicy and warm dried ginger can promote blood circulation. Modern experimental research also confirms that dried ginger has biological activities such as antiplatelet aggregation and anticoagulation [57]. Experimental study showed that the total effective rate of dried ginger liniment for the treatment of chapped hands and feet was 88.6%, which was higher than 68.0% of the control group. The reason was that dried ginger contained spicy ingredients such as volatile oil, which could promote local blood circulation and play a role in protecting the wound and promoting healing. In addition, the water extract and volatile oil of dried ginger have been found to have effects in preventing thrombosis and inhibiting platelet aggregation [58].

It is also reported that dried ginger can protect the liver and regulate metabolism and other pharmacological activities. The water extract of dried ginger can reduce the level of malondialdehyde (MDA) in rats with acute liver injury induced by acetaminophen, and enhance the activities of antioxidant and free radical scavenger glutathione (GSH) and superoxide dismutase (SOD), effectively weaken acetaminophen hepatotoxicity and improve the degree of hepatocyte necrosis [59]. Kucukler et al. [59] studied the protective mechanism of zingerone on VCM-induced liver toxicity in rats and found that zingerone could significantly reduce the levels of three inflammatory factors (NF-κB, tumor necrosis factor (TNF-α) and interleukin-1β (IL-1β)) in rats with liver toxicity, and reduce the activity of nitric oxide synthase (iNOS) and cyclooxygenase-2 (COX-2) enzyme. Besides, dried ginger contains aromatic volatile oil, which slightly stimulates the digestive tract and enhances intestinal tension, rhythm and peristalsis, thereby promoting gastrointestinal digestion [60]. Zhang Guanglong et al. [61] also found that 6-gingerol had effects on the changes of gastrointestinal hormone levels in rats such as gastrin, motilin, vasoactive intestinal acid peptide, and somatostatin, and could help improve gastric motility disorder in rats undergoing chemotherapy, and concluded that its effect might be related to the regulation of gastrointestinal hormone levels in animals and the protection of interstitial cells of Cajal. Zhang Xueqiang et al. [62] found that after treating patients with antibiotic associated diarrhea (AAD) with dried ginger, it can improve the biological abundance balance and diversity of the body’s intestinal microbiota and inflammatory response, reduce the colonization of Escherichia coli pathogens, increase the content of intestinal probiotics, and reduce acute colon infection damage. The combination of multiple target experiments has good therapeutic effects on symptoms and can effectively improve the intestinal microbial environment of AAD rat.

The examination of several varieties of ginger indicates that, apart from essential elements like cellulose, glucose, and lipids, the primary components consist of volatile oils, gingerols, diarylheptanoids, and active polysaccharides. Various processing techniques lead to differences in the composition of therapeutic components. This can be observed from the specified criteria for determination stated in the “Chinese Pharmacopoeia” (2020 edition), which highlight notable alterations in specific chemical constituents of ginger and its processed derivatives [3]. Table 2 presents the various categories and modifications in the types and content of components during processing.

**Table 2:** Classification and content of chemical active components in various types of ginger based on modern pharmacological research

Processed varieties		Active ingredients [16–19]			
Volatile oils (types)		6-Shogaol	6-Gingerol	Zingerone (Y/N)	Active glycans
Fresh ginger	27	Lowest	Highest	N	Lowest
Dried ginger	27	Lower	Higher	N	Highest
Charcoal ginger	27	Highest	Lowest	Y	Lower
Roasted ginger	43	Higher	Lower	Y	Higher
Roasted ginger	Other types of active ingredients added due to processing				
	3,7,11-Trimethyl-1,6,10-Dodecantrienal				
	3,9(11)-Diene-10-peroxide				
	Cubebene				
	Eucalyptadiene				
	β-Bisabolol				

Y represents the presence of the component, N represents the absence of the component

**Fig. 3:**
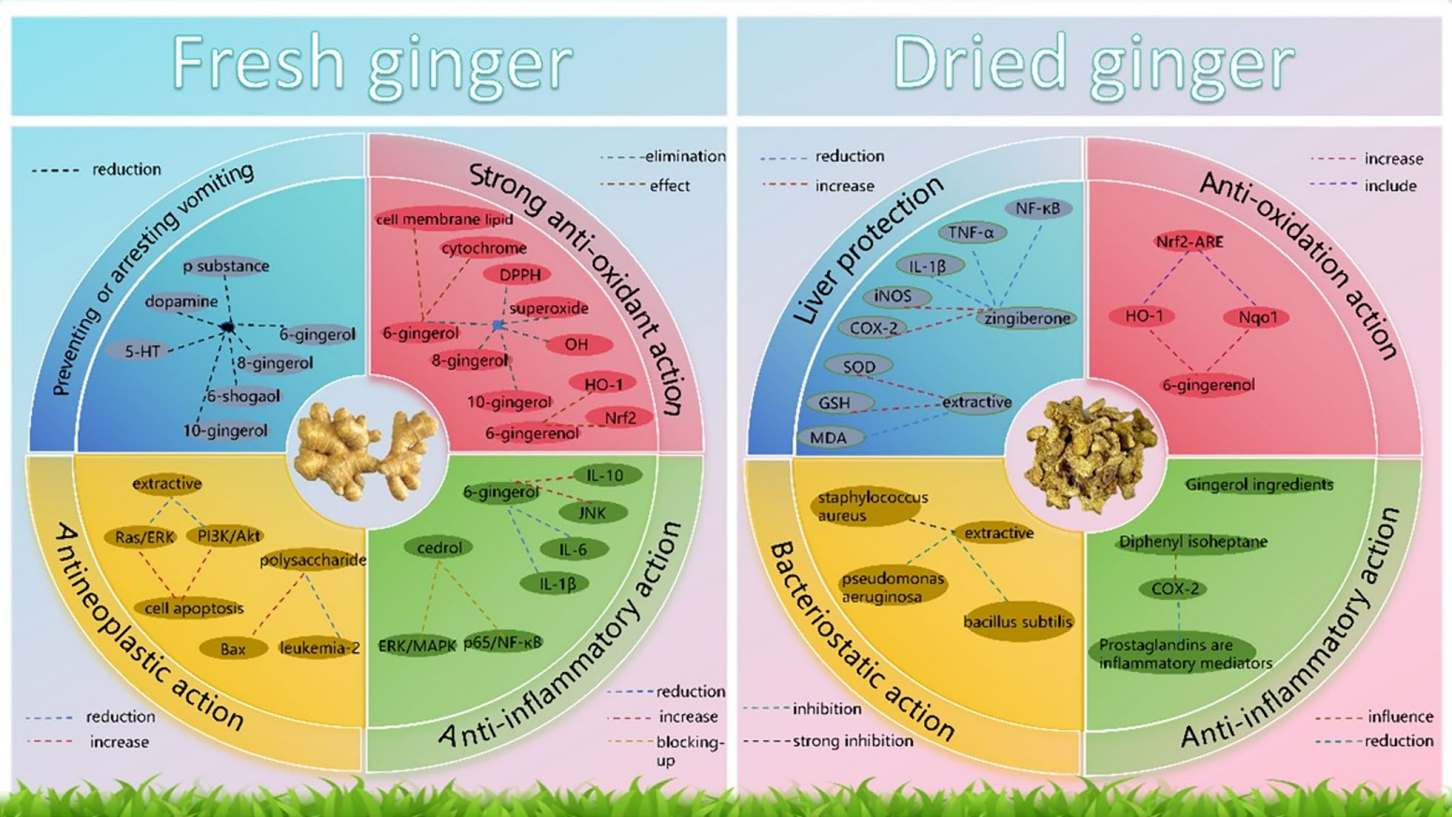
The connection and difference between the chemical composition and pharmacological effects of fresh ginger and dried ginger

As illustrated in Fig. 3, fresh ginger and dried ginger are two main subjects of pharmacological research on ginger. Most processed products are based on fresh ginger, and variations in the chemical components lead to differences in their medicinal effects, either in terms of intensity or subtle changes resulting from the addition or reduction of chemical constituents. By combining the principles of “identifying properties by effects, homology of properties and effects” with existing scientific research, an interplay and correlation between their chemical components and pharmacological actions can be observed.

Fresh ginger and dried ginger share similar chemical components that possess antioxidant and anti-inflammatory effects. However, fresh ginger exhibits a greater number of antioxidant targets and stronger effects, demonstrating its potent antioxidant activity. Additionally, fresh ginger is particularly effective in treating vomiting and inhibiting tumor activity, while dried ginger excels in protecting the liver and exerting antibacterial effects.

### Pharmacological synthesis and research status of baked ginger and other processed products

At present, there is limited modern pharmacological research on less well-known ginger processed products such as baked ginger, ginger charcoal, and roasted ginger, with roasted ginger being particularly scarce. After review, a small amount of research has shown that baked ginger has pharmacological activities such as anticoagulation, anti-inflammatory, anti-tumor, and antioxidant properties. Ginger charcoal has pharmacological activities such as warming menstruation, hemostasis, antioxidant, anti-inflammatory, and analgesic properties. For baked ginger, it was reported [63] that the water decoction of baked ginger could significantly shorten the bleeding time and clotting time of mice, while the water decoction of ginger charcoal could only significantly shorten the bleeding time of mice. In addition, the suspension of baked ginger, ginger charcoal, ether extract and water decoction all had a significant tendency to shorten the clotting time of mice. Secondly, the anti-inflammatory activity of baked ginger is also demonstrated [64]. The effects of dried ginger and baked ginger decoction on stress gastric ulcer, acetic acid-induced gastric ulcer, pyloric ligation-induced gastric ulcer and indomethacin-induced gastric ulcer in rats were studied. The results showed that the decoction of baked ginger had obvious inhibitory effect on stress gastric ulcer, acetic acid-induced gastric ulcer and pyloric ligation gastric ulcer in rats, but it was ineffective on indomethacin gastric ulcer; However, dried ginger has no such activity, which may be due to the fact that some water-soluble chemical active components of dried ginger can only be separated out after processing. In addition, studies have also found that the decoction of baked ginger and its medicinal rabbit serum have inhibitory effects on the in vitro growth of gastric cancer SGC-7901 cells and lung cancer A549 cells [65]. Its induction of tumor cell apoptosis and intervention in cell proliferation cycle may be the mechanism of its anticancer effect.

Ginger charcoal’s hemostatic activity was unique compared with that of ginger. At present, some scholars have found, using metabonomics technology, that ginger charcoal can enable the disordered endogenous biomarkers of rats with hemorrhage due to deficiency cold to return to the normal level, and identified tryptophan, glycine and lactic acid as the biomarkers for the warm-meridian hemostatic effect of ginger charcoal, revealing the warm-meridian hemostatic effect of ginger charcoal on the whole [66]. Secondly, relevant studies have found that the water decoction of carbonized ginger has a significant inhibitory effect on stress induced gastric ulcers and acetic acid induced gastric ulcers in rats, indicating that dried ginger charcoal may have anti-inflammatory effects. Some scholars also used the mouse hot bath tail flick method and hot plate method to measure the pain response time of mice, and found that the nano components of ginger charcoal have good analgesic effects on pain caused by mice in the hot bath and hot plate models [67]. In addition to the above pharmacological activities, related studies have found that different single components in ginger charcoal may have different pharmacological effects, that is, ginger charcoal may also have various physiological activities such as anti-tumor, anti-endocrine disorder, anti-pathogenic microorganisms, anti-vomiting, anti-nerve injury and cell protection. However, due to limited clinical experimental research on ginger charcoal and limited data available for reference, further basic research on the pharmacological effects and clinical applications of ginger charcoal should be carried out to further improve its pharmacological effects and clarify the mechanism of its characteristic hemostatic pharmacological effects.

## Study on clinical application of different kinds of ginger

### Fresh ginger is good at moving around in the body, alleviating vomiting and relieving exterior syndromes

Fresh ginger is clinically used to treat common cold due to wind-cold syndrome, stomach cold vomiting, cold phlegm and cough, poisoning from fish and crabs, etc. “Treatise on Febrile and Miscellaneous Diseases” contains 269 prescriptions, of which 113 were made with ginger, and 67 were made with fresh ginger. Zhang Zhongjing uses fresh ginger the most in the chapter Tai yang disease. What is the Tai yang? It is a large amount of yang energy. Tai yang, also known as the giant yang, has a strong yang energy. It serves as a barrier for various meridians and also controls the Ying qi and Wei qi, with the role of the camp outside the Wei qi. If the evil of wind and cold attacks the exterior, the sun is the first to bear the brunt, presenting a wind and cold exterior syndrome [68]. In “Treatise on Febrile and Miscellaneous Diseases”, the efficacies of fresh ginger in warming spleen and stomach for dispelling cold and stopping vomiting are used in such prescriptions as Dachaihu Decoction, Zhizi Shengjiangchi Decoction, Xuanfu Daizhe Decoction, and Shengjiang Xiexin Decoction. A large amount of fresh ginger is used in Dachaihu Decoction to stop vomiting. Fresh ginger has significant effects on normalizing stomach by guiding qi downward, and warming the Middle-jiao to arrest vomiting. Its clinical application is extensive, and it can be used in combination with various causes of vomiting. Those with mild vomiting symptoms can be treated with Shengjiang Dazao Decoction, while those with severe vomiting can be used in combination with Pinellia ternata; Xiaobanxia Decoction can be used for stomach cold and vomiting. Fresh ginger and Pinellia ternata can be combined to disperse pungency and pathogens, regulate stomach qi, normalize stomach by guiding qi downward, and warm the Middle-jiao to arrest vomiting; Vomiting caused by cold fluid can be treated with Shengjiang Banxia Decoction or Juzhijiang Decoction; Liver qi stagnation and heat transformation, disharmony of Shaoyang qi, and vomiting caused by Middle-jiao can be treated with Bentun Decoction; Spleen stomach deficiency cold-caused vomiting can be treated with cinnamon twig and peony, such as Xiaojianzhong Decoction; Stomach heat-caused vomiting can be treated with stomach clearing and antiemetic drugs such as Huanglian and Zhuru. Fresh ginger is also good medicine for treating stomach cold and vomiting. Patients with chronic gastritis are prone to vomiting after their stomach gets cold. Fresh ginger is pungent and mild in nature, and it can warm the Middle-jiao to arrest vomiting. It can eliminate the cold pathogen of Middle-jiao by warming the interior. Fresh ginger can also be used to treat vomiting due to stomach deficiency, vomiting due to stomach yin deficiency, gastric regurgitation and vomiting due to pregnancy. In addition, fresh ginger is also used in clinic for febrile wind, cold phlegm cough or spleen stomach deficiency cold, abdominal cold pain, appetite loss, nausea and vomiting. Its anti-emetic effect is also widely used in modern clinical practice. Fresh ginger can treat nausea and leaf emesis from pregnancy, and relieve the acute nausea symptoms of patients receiving carboplatin paclitaxel combination therapy [69]. In addition, fresh ginger can relieve the drug poisoning of unprocessed Pinellia ternata, Arisaema consanguineum Schott and other food poisoning of fish and crab. It also has antioxidant and bacteriostatic effects, and is often used as an antioxidant and preservative, which plays an important role in food and other chemical industries.

### Dried ginger has a wide range of properties, can warm the interior and return yang

Dried ginger is clinically used to treat abdominal cold pain of spleen stomach cold syndrome, Yang depletion syndrome and cold drink asthma cough. In the ancient “Treatise on Febrile and Miscellaneous Diseases”, a total of 43 prescriptions used dried ginger in 113 prescriptions, of which 8 prescriptions for the symptom contained “vexation”. Vexation refers to the self-conscious symptoms of vexation and anxiety in the heart, which are mostly seen in heat syndrome [70]. Fresh ginger can “unblock the minds” and dried ginger can also control it. According to the original text of “Shennong's Classic of Materia Medica”, “the living is especially good”. Zhang Zhongjing, the medical saint, deeply understood its purpose and drew inferences from one instance. He used the effect of ginger to “unblock the minds” to treat the trouble of disturbing the mind due to the extreme deficiency of Yang qi, which can not be depressed, and the deficiency of Yang has a good effect of clearing the mind and eliminating the trouble. Ganjiang Fuzi Decoction, Baitong Jia Zhudanzhi Decoction, Fuling Sini Decoction, Chaihu Guizhi Ganjiang Decoction, Zhizi Ganjiang Decoction, Gancao Ganjiang Decoction, Gancao Xiexin Decoction, and Wumei Pill are made for irritating heat. None of the eight prescriptions listed here has excess heat. Taiyang syndrome is mistakenly treated to hurt Yang, and the pulse sinks and the limbs are faint. For example, Ganjiang Fuzi Decoction and Gancao Ganjiang Decoction are used. It is also used by people who are injured by Taiyang syndrome and have mixed cold and heat to form ruffians in Banxia Xiexin Decoction and Gancao Xiexin Decoction. In fact, the syndrome Ganjiang Fuzi Decoction applies to is only the Taiyang syndrome deficiency and internal cold due to excessive sweating, which leads to Yang deficiency in both the exterior and interior, and is combined with fluid and blood damage. Ganjiang Fuzi Decoction can be used as a prescription for the treatment of general Yang deficiency and internal cold, and it cannot be considered as an agent for the treatment of severe Yin cold [71]. Dried ginger is used for Shaoyin disease in Shaoyin cold syndrome, and it is compatible with aconite. The two must be used to help Yang disperse cold to restore Yang and stem counterflow by using decoction such as Sini Decoction and Baitong Decoction. Dried ginger is also used to treat vomiting due to Zhongyang deficiency, by use in medicine such as Lizhong Pill. Dried ginger is also used for cough by using decoction such as Xiaoqinglong Decoction and Linggan Wuwei Jiangxin Decoction. In addition, the curative effect of Ganjiang Xiaochaihu Decoction in the treatment of mild patients with Covid-19 Omicron variant infection is satisfactory, which can effectively improve the patients’ TCM syndromes, relieve depression and anxiety, and has the effect of “treating both disease and depression”.

### Baked ginger is divided into blood phases to warm meridians and stop bleeding

In the Synopsis of Golden Chamber, the prescription of baked ginger is Gancao Ganjiang Decoction. This baked ginger is used to treat the deficiency and cold in the lung, and for those who cannot take the body fluid, its meaning of warming and convergence is taken. Baked ginger can still warm the spleen and stomach, relieve pain and stop diarrhea. For example, Erjiangyuan is used to “nourish the spleen and warm the stomach, remove cold and eliminate phlegm”. “Zhengzhi Zhunsheng Nuke”(1602) recorded the treatment of deficiency cold in the Middle qi with baked ginger [72]; Duanxia Decoction is used to “treat Chong Ren Qi deficiency, collapse and leakage”. Baked ginger is widely used in gynecological diseases. “Fuqingzhu Nuke” [73] contains more than 170 prescriptions, including more than 20 prescriptions of baked ginger (black ginger), which are scattered in the chapters of blood avalanche, parturition, postpartum, etc. Its mechanism of action is summarized by Yin Xianghua et al.[74] as follows: guiding blood to meridians, supplementing fire of vital gate and astringing treatment of blood avalanche; activating blood circulation to remove blood stasis, warming the interior and stopping bleeding to treat miscarriage; removing blood stasis and generating new blood, warming the Middle-jiao and dispersing cold for postpartum treatment; warming the Middle-jiao and restoring Yang, removing blood stasis and hemostasis, and removing blood dizziness.

### Ginger charcoal has good retention and is good at warming meridians and stopping bleeding

According to relevant research reports, ginger charcoal is often used in the clinical treatment of digestive system diseases, gynecological diseases and other diseases. Ginger charcoal is warm in nature. It can remove the cold in the stomach and keep the interior. It can convergence and stop diarrhea and dysentery. It can be used to treat digestive system diseases. In clinical practice, ginger charcoal is used to treat ulcerative colitis by tonifying the spleen and kidney, dispelling lower-jiao blood stasis and astringent effects. Ginger charcoal is used to warm the interior and strengthen the spleen, warm the Middle-jiao and strengthen the spleen, and treat chronic diarrhea. Ginger charcoal is used in combination with platycladus orientalis charcoal, prunus mume charcoal, mugwort leaf charcoal, and crinis carbonisatus to treat asthenic cold hematemesis, blood defecation, etc. Ginger charcoal acts on the liver and spleen meridians, which is specialized in warming the meridians and hemostasis, and can be used to treat gynecological diseases [75]. In clinical practice, Yanghe Decoction is used to treat breast hyperplasia, endometriosis and chronic pelvic inflammatory disease with its effects of warming Yang and tonifying blood, dispersing cold and activating stagnation. Fuyuekang capsule, a Chinese patent medicine preparation, can be used for patients to alleviate postpartum lochia or less abdominal pain, and reduce the amount and time of bleeding after drug abortion. It has the effects of activating blood circulation, removing blood stasis, and relieving pain. Sometimes in clinical practice, ginger charcoal is often used instead of baked ginger in order to strengthen the function of astringency and hemostasis. In addition, Professor Tang Xudong [11] used Xiaoyao Powder addition and subtraction. The ginger charcoal in the prescription mainly enters the blood. The pungent nature is lost, and the drug has a wide range of properties and rapid action. The heat is less than that of baked ginger. It has the effects of soothing the liver and spleen, regulating cold and heat, and tonifying the intestines is the best. Related reports [76] have shown that the combination of Fuzi Lizhong Decoction and Shenling Baizhu Powder has the effect of warming the Middle-jiao and dispersing cold, with the use of baked ginger charcoal in the formula. The effect is gentle and long-lasting, making it very suitable for prolonged diarrhea due to deficiency cold. Professor Ban Xiuwen [77] transformed the Zhigancao Decoction into a mixture of ginger charcoal with a warm and astringent nature, and used it in combination with motherwort and cinnamon to replenish Qi and nourish Yin, replenish blood stasis, and stop leakage, producing its effect of resolving blood stasis without moving blood, stopping leakage without leaving blood stasis.

### Roasted ginger has a pungent taste and warm nature, and it can be used to stop vomiting and diarrhea

The nature of roasted ginger is neither as loose as fresh ginger, nor as dry as dried ginger. Therefore, it slightly integrates the advantages of both. It is good at “treating stomach cold, diarrhea, and acid swallowing”, and therefore has a special focus on the spleen and stomach [78]. The New Compilation of Materia Medica further proposes the indications and compatibility of this medicine: “Roasted ginger can regulate the middle warmer and stop vomiting, unlike fresh ginger which is too loose and or dried ginger which is too dry. Therefore, only the roasted ginger can avoid these problems. It is most safe to regulate the middle warmer and stop vomiting and use it in combination with jujube, and to mix it with the body fluids of the spleen and stomach, and to regulate Ying qi and Wei qi”. Li Zhongzi [79] used Liujunzi Decoction to remove poria cocos, add astragalus membranous, and roasted ginger to treat fever and headache caused by anxiety, spleen deficiency, and weak Qi. Xu Jingshi [80] used a formula containing roasted ginger to treat recurrent abdominal pain and diarrhea due to wind cold in the stomach caused by a history of gastric polypectomy surgery; it is gastrointestinal damage caused by postoperative chemotherapy in elderly patients with colon cancer, resulting in Qi deficiency and disheartening, and damage to both Qi and body; During the active phase of chronic non atrophic gastritis, symptoms include Qi reflux and phlegm obstruction, acid reflux and belching due to cold, and frequent hiccups. It can be seen that roasted ginger can soothe the stomach and stop vomiting. It is gentle and effective, and is more suitable for cancer patients with weakened systems. There are reports that in clinical observation and nursing, it has been found that Weijiang Decoction can prevent and treat gastrointestinal adverse reactions caused by chemotherapy, and can greatly improve the quality of life of cancer patients. In addition, the “Benjing Fengyaun” states “When fresh ginger is roasted, it falls but does not rise, relieving abdominal pain and relieving diuresis, supporting the spleen, and dispersing stagnation, so Xiaoyao Powder is used.” Xiaoyao Powder (Pill) containing roasted ginger ingredients is widely used to treat gynecological diseases such as dysmenorrhea, metrorrhagia and premenstrual breast distension, digestive system diseases such as functional dyspepsia, chronic gastritis and intestinal stress syndrome, ophthalmic diseases such as glaucoma, dry eye disease and vitreous opacity, mental illnesses such as depression, schizophrenia, reactive psychosis and neurasthenia, as well as melasma.

## Discussion

### Differences and advantages of different kinds of ginger

**Fig. 4:**
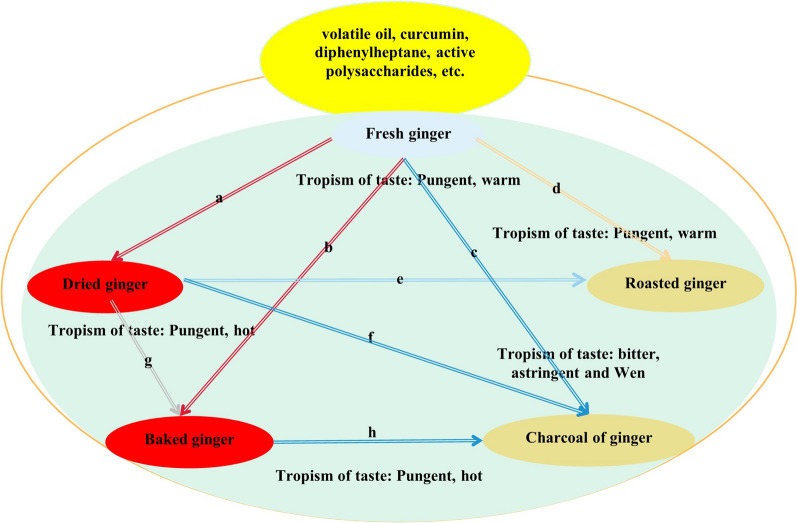
The medicinal and taste properties of various ginger. **a** Positive enhancement, reviving yang; **b** Positive enhancement, warm the middle warmer; **c** The taste turns bitter and stops the bleeding; **d** The dispersion is weakened; **e** The dryness is weakened; **f** Stir-baked the crude drugs into black on outside and brown in inside; **g** Less volatile oil; **h** Yin and Yang turn, the main function is hemostasis

As illustrated in Fig. 4, despite the continuous changes in TCM culture, various kinds of ginger still have official records to distinguish their medicinal use, without being screened out. Each has its own unique medicinal value. From this study, it can be seen that various ginger varieties show differences or their own advantages in the properties, taste, meridian tropism, efficacy, and clinical application. Reasonable differentiation should be made among them in clinical applications in order to give full play to therapeutic effects. Fresh ginger is spicy and slightly warm, leading to dispersion, and is good at moving around the body without clinging to a specific location, good at dealing with wind-cold, flu and sweating, regulating the middle warmer and stopping vomiting; Dried ginger is pungent and hot, is good at moving around the body while clinging to a specific location, and good at warming the Middle-jiao and restoring yang. It is suitable for those with excessive cold and dampness in the Middle-jiao, as well as for those with asthma and cough caused by cold drinking and depression in the lungs. Moreover, due to its strong effect, it is very effective in restoring yang and relieving adverse reactions. Both baked ginger and ginger charcoal are processed products of dried ginger. Baked ginger is pungent and hot, with the intention of removing some volatile oils. Therefore, its medicinal properties are not as good as that of dried ginger, and its heat is no less than that of fresh ginger. The power of warming inside is not as strong as that of dried ginger, so it is more inclined to warming the Middle-jiao; Ginger charcoal is bitter, astringent, and warm. Its processing is intended to stir fry the charcoal to preserve its properties. Compared to fresh ginger, dried ginger, and baked ginger, its bitterness disappears and remains unchanged. Its warming effect is weak, and its astringent effect is strong. It is better at stopping bleeding and diarrhea. For roasted ginger, it is pungent and warm in nature, not as warm as fresh ginger, and not as dry as dried ginger. It is gentler when used, and it’s warming the Middle-jiao and prevent vomiting is enhanced in moderation. It is more suitable for people with weak physical constitution and has good therapeutic effects. According to the corresponding clinical application analysis and comparison in this paper, it can also be seen that each ginger has its own preference for efficacy. Therefore, the current clinical application of various kinds of ginger should be differentiated based on the specific symptoms of patients, which is also the value of each ginger.

### Holistic view and one source and multiple substances

The property of TCM is an outstanding product of the combination of ancient Chinese medical practice and philosophy, which highly summarizes the performance and efficacy of TCM, in which the nature and taste reflect the effect of drugs on the pathological bias of human body cold and heat, the rise and fall of Yin and Yang, and the action characteristics of drugs such as reinforcing, reducing, dispersing and converging; According to the theory of Zang Xiang and meridian in TCM and the location of the disease according to the treatment of drugs, the meridian tropism reflects the selective effect of drugs on a certain part or some parts of the body; The rise and fall reflect the action trend of drugs, and the toxicity reflects the safety degree of drugs; Efficacy is a special expression of the therapeutic effect of drugs on human body, which is summarized through clinical practice under the guidance of TCM theory. It highly summarizes the effects of drug prevention, diagnosis, treatment and health care, and has typical features of TCM. In a word, the property of TCM is aimed at the whole TCM itself, highly integrated in connection with the clinical therapeutic effect, and has a very strong holistic view. “Recognition of effectiveness on nature, homology of nature and effect, and mutual expression of nature and effect” is the most intuitive description. Different from the cognition of the properties of TCM, modern research on TCM starts from the main chemical components of TCM, analyzes the pharmacological activities of a single or a class of chemical components, expands the pharmacological effects of TCM on the basis of TCM theory, simplifies the research on TCM, and endows the feasibility of modern research on TCM. However, it has abandoned the holistic view of TCM properties and ignored the mutual influence and balance restriction of multiple chemical components when they act simultaneously in the body.

However, it also limits the research of TCM from one source and multiple substances with medicinal value gradually fading out of the global vision, which is not conducive to the modernization of TCM and the construction of massive health. The most intuitive manifestation is that the main chemical components of all ginger are almost the same, and the difference lies in the different content of the main chemical components and the individual chemical components added after processing, which leads to nearly the same pharmacological activity of all ginger. In general, all ginger can be substituted for each other in clinical use, but in fact, the clinical medicine of all ginger has its own characteristics and advantages, and the pure chemical component research has obliterated the medicinal value of all ginger in disguise. Therefore, in the new era of drug research, TCM research complies with the times to carry out pharmacological activity analysis based on chemical components, but the “holistic view” of TCM itself cannot be ignored. The properties of TCM still play an important guiding role in the research of TCM modernization, fundamentally affecting the research of TCM with one source and multiple substances.

### Analysis on global research progress of ginger

Ginger has a long history in the world, especially in China. In addition to the food field, it still has a deep background in the field of TCM in China, and has a broader application prospect in today's era of massive health. Over the years, fresh ginger has been the focus of attention for people in the world, while other ginger products have been rarely studied due to their similar efficacy. In fact, as a typical case of TCM with one source and multiple substances, ginger can have its nutritional value and different medicinal effects increased whether through different planting methods or through a series of different processing. In order to help the world's scientific researchers have a clearer understanding of the application development and research of ginger, from the perspective of patent application, studies of it in the field of drugs and the economic benefits generated are analyzed in this research. According to incomplete statistics, more than 75 countries in the world are involved in the research of ginger, among which China have conducted the most research, with as many as 60,000 invention applications, followed by South Korea, the United States, Japan and other countries. Among all patent applications, there are 45,456 in A23 category, 28,929 in A61 category and 3,481 in A01 category. Among them, category A61 is medicine, veterinary medicine or hygiene related patent applications. Further analysis of them revealed that Chinese invention applications accounted for a large proportion, with 19,971 patents. Through the analysis of A61 related patent application trend and technology life cycle, it is found that the research on ginger showed an increasing trend from 2000 to 2015, but gradually decreased after 2015, and its research heat has decreased. Further research on the patents of ginger was carried out separately, and it was found that except for fresh ginger and dried ginger, the patents and research of baked ginger, ginger charcoal and roasted ginger were relatively rare, and they were mainly concentrated in China, and the number of relevant researches in other countries was very small. According to analysis, it is speculated in the research that the reason is that foreign scholars lack knowledge about TCM theory and have little knowledge about the processed products of ginger. Lack of research on ginger processed products at home and abroad is also one of the reasons why the research heat has been declining in recent years.

## Conclusion

From the perspective of one source and multiple substances, according to TCM theory and modern pharmacological activity research, the analysis of various kinds of ginger was conducted from the aspects of source, performance, efficacy, and clinical application. It was found that there are differences and advantages in various kinds of ginger from their performance, efficacy, to clinical application. Fresh ginger is good at moving around in the body, stopping vomiting and relieving exterior syndromes; Dried ginger not only disperses cold in a warm state, but also stops diarrhea in a warm state, growing in the warm and returning back to yang; The dryness nature of baked ginger is not as good as that of dried ginger, and it can relieve pain, bleeding, and diarrhea through warming the Middle-jiao; The carbonizing processes of ginger charcoal and stir fried charcoal have good retention and are good at warming meridians and stopping bleeding; The pungent and dispersing power of roasted ginger is not as strong as that of fresh ginger, and its dryness is not as high as that of dried ginger. Its medicinal properties are mild and it is good for weak patients to stop vomiting, mainly acting upon the middle. However, according to research, it can be seen that the pharmacological activity analysis results of various kinds of ginger are not significantly different. Due to the same basic elements of multiple traditional Chinese medicines, their main chemical components are almost the same, and only changes in the content, additions, or deletions of chemical components occur during different processing processes. Therefore, based on the study of chemical components, the pharmacological activities of various kinds of ginger have antioxidant, anti-inflammatory, anti-tumor, antibacterial, anticoagulant effects, etc., and it is not possible to fully understand the clinical medicinal value of various kinds of ginger. This also limits the world’s understanding of TCM to a certain extent, resulting in the scarcity of research on ginger at home and abroad.

One source and multiple substances of TCM have certain particularity. The study of pharmacological activity based on the main chemical components alone cannot explore the respective medicinal values of different kinds of ginger, and reasonably and scientifically guide clinical medication. Therefore, based on the perspective of one source and multiple substances, the research on the modernization of TCM must start from TCM theory, integrate the holistic view of TCM theory, correctly understand the “recognizing nature by efficacy, homology and mutual expression of nature and efficacy”, utilize modern drug research technology to build a bridge for the modernization of TCM, and do a good job in the development of the world's massive health cause.

## Data Availability

Not Applicable.

## Abbreviations

DPPH: 1,1−Diphenyl−2−picylhydrazyl
MDA: Malondiadehyde
GSSG: Oxidized glutathione
GSH: Glutathione
HO−1: Heme oxygenase−1
ROS: Reactive oxygen species
STAT3: Signaltransducers and activators of transcription 3
PT: Prothrombin time
ATPT: Activated partial thrombin time
SOD: Superoxide dismutase
AAD: Antibiotic associated diarrhea
